# Probabilities

**Published:** 1875-10

**Authors:** G. V. Black

**Affiliations:** Jacksonville, Ill.


AMERICAN JOURNAL

OF

DENTAL SCIENCE.

Vol. IX. THIRD SERIES—OCTOBER, 1875. No. 6.

### ARTICLE I.

### *Probabilities.*

BY G. V. BLACK, JACKSONVILLE, ILL.

Read before the Illinois State Dental Society.

How can we best know the probable result of operations in the varied and complex conditions in which patients present themselves? It is patent to every one that if in any case we should fail to determine this point with a reasonable degree of definiteness, we will be operating more or less at random. Such a course of procedure cannot be too strongly condemned. This is perhaps no easy question to solve; indeed, cannot always be correctly solved by the most able among us. But the degree of our knowledge of the cases presented, and the extent and accuracy of our own observations, combined with a just estimate of our own skill in manipulation, must always be our principle dependence in arriving at correct conclusions.

There are, however, some considerations outside, so to speak, of the dental school and the text-book, that it may be well for us to examine. We cannot always adopt that plan which the pathology of the special case seems to indicate, and by pursuing it rigidly do the best thing for our patient.

There are often surrounding and trammeling circumstances, not at all of a pathological nature, that must be taken into consideration if we would correctly estimate the probabilities of the case. These incidental conditions, or circumstances, accompany in some degree almost if not every patient that presents himself, and are as various as the persons; and it not unfrequently happens that it is more difficult to correctly define and estimate these surrounding conditions than to determine the pathological conditions and estimate their requirements.

I know of but one general rule that is of universal application, and that is to examine and treat each case with an earnest purpose to do that which will most benefit them under existing conditions and circumstances. If we do this we must necessarily learn something of the patient's habits and mode of living. We indeed learn to know, so to speak, many of our patients as soon as we look at them, but we cannot thus form a judgment that will always be well founded; and it is almost always best, if the patient by a stranger, to ask judicious questions as to their mode of living, their habits and general health, and their estimate of the value of their teeth, &c., and skillfully lead them to talk of themselves so that we may know something as to what there future course will be, and also as to what forms of disease may be exerting their influence upon the teeth, if diseased conditions exist. We will often find it best to extract teeth for one patient, and treat and fill for another, where the condition of the teeth themselves are precisely similar, owing to differences in health or habits. For instance: suppose a young lady presents herself with a large number of decays, in active progress, with a number of pulps exposed and giving trouble. After talking with her you find that she is of irregular and careless habit, and that her principal thought is relief from present suffering; and the conversation fails to elicit any satisfactory assurance that if the case is properly treated now, it will be judiciously followed up in the future—could we properly estimate the probable results of treatment as the same, as in a precisely similar case of decay, where the patient had a lively appreciation of the value of her teeth, and showed by word, and act, that our efforts would be seconded and future treatment followed up on her part? Certainly not? And it will often be best for our patient that we vary the treatment accordingly, removing perhaps a number of teeth for our careless patient, but being careful to reserve, if possible, those that are so situated, that all will be brought into active use in mastication. This is a point worthy of special thought. Where a patient will take no great care of her teeth, if we can reserve from the wreck of the molars and bicuspids a few that will serve the purposes of mastication and throw all the work upon them, this employment of them will do much to insure their preservation, and the probabilities of the case will be much better than they would be if we endeavored to preserve a greater number. I have, as I think, often found very great advantage from pursuing this course. It may sometimes call for the filling of some difficult cases, and the extraction of teeth less decayed, which at first sight might not seem to be justifiable but the sequel will generally prove it to be good practice. It may be urged, and properly too, that it is the business of the operator to overcome this carelessness in regard to the care of the teeth, by judicious instructions and advice. In common with our brother practitioners, we have essayed to do this with very varying results, many times accomplishing great good, but in most instances, I fear, that the probabilities of lasting good from this source is, to say the least very doubtful. We should not, however, cease to try it because we often fail. We can usually do most by showing by word and act that we are earnest, and careful, as to our patient's welfare, and carefully leading them to understand the necessity that our efforts be seconded and followed up by them, that they must take their part of the labor to insure the best results of treatment.

Failure of appreciation, while it is the most aggravating is not the only thing we have to contend with. Many persons who will gladly do all in their power are so situated that they cannot present themselves as the proper treatment of their case demands, and from this cause the probabilities of good from this or that course of treatment may be much diminished. It becomes the duty of the operator to search out these drawbacks and endeavor to pursue that general plan which gives the strongest probabilities of good results under these disadvantageous circumstances; remembering always, that the greatest part of labor we perform is for the future, rather than the present, and immediate good of our patient.

We are often called upon to treat for children; first molars that are not very largely decayed perhaps, but with exposed or abscessed pulps, or with pulps in a seriously inflamed condition, so that our best endeavors will fail to restore them to health. Now we can treat these teeth successfully for the present by removing the remains of the pulps and filling the roots. But what are the probabilities as to their endurance? My experience is that they will generally become brittle and break away after a few years, and finally be lost. The dentine seems to lack those properties which we find later in life which render it strong and enduring after the death of the pulp. Then the probabilities are that they cannot be permanently retained. Then shall they be extracted at once? Before this qnestion is decided the entire mouth and face should be closely examined, if we would do the best thing possible for our patient. Is it *one* of the first molars in this condition, or is it a majority of them?

We will first suppose it is but one that has lost or must lose its pulp. All the others, if decayed, can be treated with a reasonable probability of saving their pulps in a healthy condition and consequently of retaining the teeth permanently. The other teeth promise well while the development of the features is regular and symmetrical. Now if we treat and fill this tooth, the probabilities are that it will begin to break away after a few years, become a source of trouble, and be finally lost. If we extract it, the probabilities are that more or less distortion of the features will result from its loss. The space of the lost member will be filled up by the loaning in toward it of the remaining teeth ; and in this way the anterior teeth will be carried to one side of the mediary line, forever marring their appearance, more or less, every time the lips are parted,—robbing a pleasant word or smile of much of its wonted charms. The lips and wing of the nose are also in some degree carried with the teeth. The longer this tooth is retained the less the distortion. We therefore unhesitatingly say, retain the tooth, if possible, until the anterior teeth have become firmly fixed in their position. It must also be remembered that it is one office of the sixth-year molars to hold the jaws in position while the temporary teeth are being shed and the permanent teeth are taking their place. They are four large and strong teeth, taking their places just in such position as to antagonize with each other and receive the strain of the powerful masseter and temporal muscles. Now if we remove one of these teeth any considerable time before the other permanent teeth have taken their places, the probabilities are that the continued strain of these powerful muscles being unmet by the usual and proper resistance, will bend that side of the inferior maxilla or draw it out of its proper position more or less, at a time of life when this bone is in an active stage of growth, producing an irregularity and deformity of the features which will be retained in after-life.

If we remove the case to the lower jaw and suppose it to be one of the lower molars that must loose its pulp, the probabilities of distortion of the features from the effects of the muscles are the same, while the same results will be seen in the lower incisors as we have pointed out in the upper; the case therefore requires the same consideration and treatment.

Now let us suppose that all, or a majority of the sixth-year molars, must lose their pulps. If we remove them there is no particular danger of distortion from side to side, Yet here again we must study the probable effects of their removal, for all these spaces will fill up largely at the expense of the prominence of the mouth. Will the dental arch probably be so prominent as to suffer the loss from shortening without marring the features, or will it lack prominence at best and the shortening of the arch from the loss of the molars seriously near the features in after-life; or again, is the arch inclined to be prominent? Upon the determination of these questions we should base our treatment. If it is decided that the arch should be kept as full as possible the teeth should be retained until the anterior teeth have become fixed in their positions. If the arch will probably be too prominent, extraction will be indicated. In case of the loss of the pulps in both the first molars of the upper jaw, their extraction or retention should be determined by the probable comparative fullness of the two jaws. If the upper teeth will probably be too prominent, extract them; if the reverse, they should certainly be retained and receive our best care; this applies with equal force to the lower jaw.

What we have said of the first molars as to distortion from side to side applies with some force to the bicuspids when these are lost very early, but in a far less degree to the second molars; their loss has but little effect upon the position of the anterior teeth or the features. The anterior teeth that lose their pulps early, although liable to break away after a while, should always receive the best care we are able to bestow upon them. Very much may be done for young patients by searching out and correcting those conditions which point to probable decay of the permanent teeth, especially during the time of the shedding of the temporary teeth. Very many indications point to this as the time at which the foundation of many of the decays of after-life is laid. This arises in the main from the irritation of the mucous membrane about the temporary and coming permanent teeth and the consequent vitiation of the secretions by acid exudations from them, not unfrequently producing intense acidity, which excoriates and roughens the enamel upon the teeth that are taking their places, often forming cavities in a very short time, or on the other hand rendering them far more liable to be attacked in the future. Much close observation has led me to conclude that the fact that the first molars are in their places throughout the process of shedding and replacement is the principal cause of their being so much more often attacked early than the other teeth. Indeed, we believe that if the first molars can pass through the time of shedding and replacement without blemish, the probabilities as to their resistance of decay is fully as good, if not better, than any other teeth; owing probably to the fact that their situation calls them into more active and continued service in mastication. All irritations about the shedding or coming teeth, especially those that assume a low chronic form, may be regarded as probable cause of future decay among the adjacent teeth and should receive our careful attention. Its cause should be searched out and removed. As a convenient point from which to study the effects of irritation in producing decay, we would cite the profession to the wisdom teeth and ask that they take special notice of them. It will generally be found that those that come through the gums with little or no irritation resist decay about as well as any other teeth, especially if brought into active use in mastication; while those about which there is long-continued irritation are most generally attacked almost as soon as they have taken their places. Simply severe inflammation, ending in separations about the tooth and then ceasing, has no injurious effect. In all cases of filling teeth the object is, or should be, future benefit. The probable results then, of these operations, should be the basis upon which our decision as to its advisability should be founded. In most cases, perhaps, this decision will be based upon the examiation of the particular tooth upon which we propose to operate, and will depend upon the extent of the decay and the condition of its pulp and membranes. Yet in very many eases considerations of other parts must not be disregarded. The condition of the mucous membrane of the mouth may be such as to render the filling of any cavity, for the present at least, unadvisable. The same is true of all sorts of nervous affections or other pathological conditions that induce hyperæsthetic conditions of the system at large of the mucous membranes or of the teeth especially. Much experience and close observation has led me to conclude that fillings made when such conditions exist will not be so successful as they would prove if these conditions had been suffered to pass away, or had been terminated before the operation. In any and all these conditions the pulps and investing membranes of the teeth upon which we operate are more liable to take on diseased action after being operated upon, and there is much increased pain and inconvenience in the proper performance of the operation, all of which tends to lessen the probabilities as to good results. Aside from surrounding pathological conditions there are many considerations in regard to the time and circumstances of the decay that demand our attention. I am persuaded that many of us fill too many cavities; that is, we often fill cavities that probably would never injure the tooth if the operation was indefinitely postponed. On the other hand, many cavities are often left much too long without filling. To judge wisely of the probabilities of decay, its progress or non-progress in particular cases, requires much close and patient observation; and I am persuaded that it is a subject too much neglected by the mass of the profession. It seems to be the habit of many, if not most of us to fill every cavity we meet with, no matter what its size or condition. Not only that, but to cut out every spot and substitute a filling without consulting our previous observations and endeavoring to form some opinion as to the probable future of the case if left to itself, or as to whether the operation will be of royal advantage in the future. This is not as it should be, and I would urge every practitioner to make some specialty of the study of the probabilities of decay, and endeavor to establish some definite idea of the rate of progress of the various phases of decay in various conditions of the patient as they come under your observations, with the end in view of lessening the number of fillings and the discomfort of your patients without materially lessening the probabilities of good results. Very many cavities are daily met with in the teeth of patients of regular habits, the filling of which may be postponed from time to time with the simple injunction that they need watching, and the progress of the decay watched and studied without any probable danger to the welfare of the teeth; and in very many of these cases it will be found that an operation is entirely unnecessary, and that the teeth are really in better condition without the filling than they would probably have been with it,—for we believe that it is generally conceded that very small fillings are the most unreliable. I cannot here attempt to point out those classes of decay that progress rapidly, slowly, or remain stationery. That subject was treated in my paper before this Society, 1873. I must, however, say a few words in regard to buccal cavities. Farther study of these cavities and their cause has added much to confirm the views given two years ago, viz: that they are very generally caused by a peculiar irritation of the mucous membrane adjacent, and the consequent vitiation of the mucous, which is of a temporary character, though liable to frequent recurrence, but capable of being very generally controlled by friction regularly applied. In these cavities the probabilities of benefit from fillings made after its cessation or removal, are very different —the advantage being with the latter. My advice is to break away the margins of the cavities, opening them wide and institute treatment, except in cavities with exposed pulps, or those liable to become so, which should be excavated and filled temporarily. As these cavities are uniformly very sensitive when the cause of decay is in active progress and much less so after its cessation, the comfort of our patient demands our best endeavors in this direction. We have done very little of what we had in mind to do when we undertook to write this paper, The probable effects of this or that line of treatment, followed out in detail, would require an extensive work. But if by drawing your attention to this line of thought, we shall have induced any of you to make probable results and effects more of a study in the future than you have done in the past, we will have accomplished our aim.

